# A Mode of Resusitating Patients after Inhaling the Vapor of Ether

**Published:** 1847-10

**Authors:** James Robinson

**Affiliations:** Gower Street, Bedford Square


18. *A Mode of Resuscitating Patients after Inhaling the Vapor of Ether.*—Sir,—For the last week I have been using, as a means of resuscitating patients, after inhaling the vapor of ether, pure oxygen gas, with the most perfect success. To-day, I operated in nine cases on the teeth: to each patient I gave a full dose of the ether vapor, and subsequently a few inhalations of oxygen. In not one case did the patient complain of debility, and all recovered perfectly in less than a minute and a half, timed by the medical men present. I will, by your permission, in a future number of your journal, furnish the details of these and other experiments with oxygen. I remain, &c.

JAMES ROBINSON.

Gower Street, Bedford Square, March, 1847.—*Lancet.*

